# Successful Treatment with Antibiotics Alone for Infant Rib Osteomyelitis

**DOI:** 10.1155/2022/3093784

**Published:** 2022-06-08

**Authors:** Yasuaki Matsumoto, Katsuyoshi Shimozawa, Junko Yamanaka, Yukari Atsumi, Tomomi Ota, Shinji Mochizuki, Hiroyuki Shichino

**Affiliations:** ^1^Department of Pediatrics, National Center for Global Health and Medicine Hospital, 1-21-1 Toyama, Shinjuku-ku, Tokyo 162-8655, Japan; ^2^Department of Pediatrics and Child Health, Nihon University School of Medicine, 30-1 Oyaguchikami-cho, Itabashi-ku, Tokyo 173-8610, Japan; ^3^Division of Neonatology, Nagano Children's Hospital, 3100 Toyoshina, Azumino-shi, Nagano 399-8288, Japan

## Abstract

Pediatric rib osteomyelitis is a rare disease occurring predominantly in the neonatal period and early childhood and accounting for about 1% of all pediatric osteomyelitis. Compared to osteomyelitis in other parts of the body, pediatric rib osteomyelitis shows few localized findings (such as redness and swelling) and often an indolent lesion as well either of which may delay diagnosis and thus make treatment more difficult. A previously healthy one-year-old girl came to our department with a chief complaint of fever lasting for three days. She was admitted to our department to investigate her fever. At the time of admission, radiographs showed decreased permeability in the left lung field; so, we started antimicrobial therapy on the assumption of pneumonia. On the second day of admission, methicillin-susceptible *Staphylococcus aureus* was detected in the blood culture. A further, more detailed physical examination revealed some slight left anterior chest swelling. We performed a contrast-enhanced CT scan and an MRI and diagnosed her with rib osteomyelitis complicated with a chest wall abscess. She was given intravenous cefazolin for two weeks, switched to oral cephalexin for four weeks, and then recovered completely. She was treated without surgical intervention, having showed a good response to antimicrobial therapy. Osteomyelitis of the ribs in children is reported to be more common in the lower ribs and to occur more frequently in infants. In many cases, the earliest symptoms are nonspecific, so careful examination to detect any subtle abnormalities—such as swelling or mass—is of key importance for early diagnosis in infants. Regarding treatment, most cases of hematogenous osteomyelitis resolve with antimicrobial therapy alone—although surgical intervention may be required in cases of poor response to antimicrobial therapy. Therefore, early diagnosis of rib osteomyelitis through careful physical examination may reduce the chances of requiring surgical intervention.

## 1. Introduction

Pediatric rib osteomyelitis is a rare disease accounting for approximately 1% of all pediatric osteomyelitis cases [1, 2]. It occurs predominantly in the neonatal period and early childhood, making it difficult for patients to articulate their symptoms [2]. Pediatric rib osteomyelitis is more difficult to diagnose than osteomyelitis of other sites because often the earliest symptoms are nonspecific and lack local findings (such as redness and swelling) and may also present indolent lesions [3] that can further impede diagnosis. We present a case of an infant with rib osteomyelitis complicated with chest wall abscess. The patient was treated successfully with antimicrobial therapy alone and avoided surgical intervention due to early, careful physical examination.

## 2. Case Presentation

**Figure 1:**
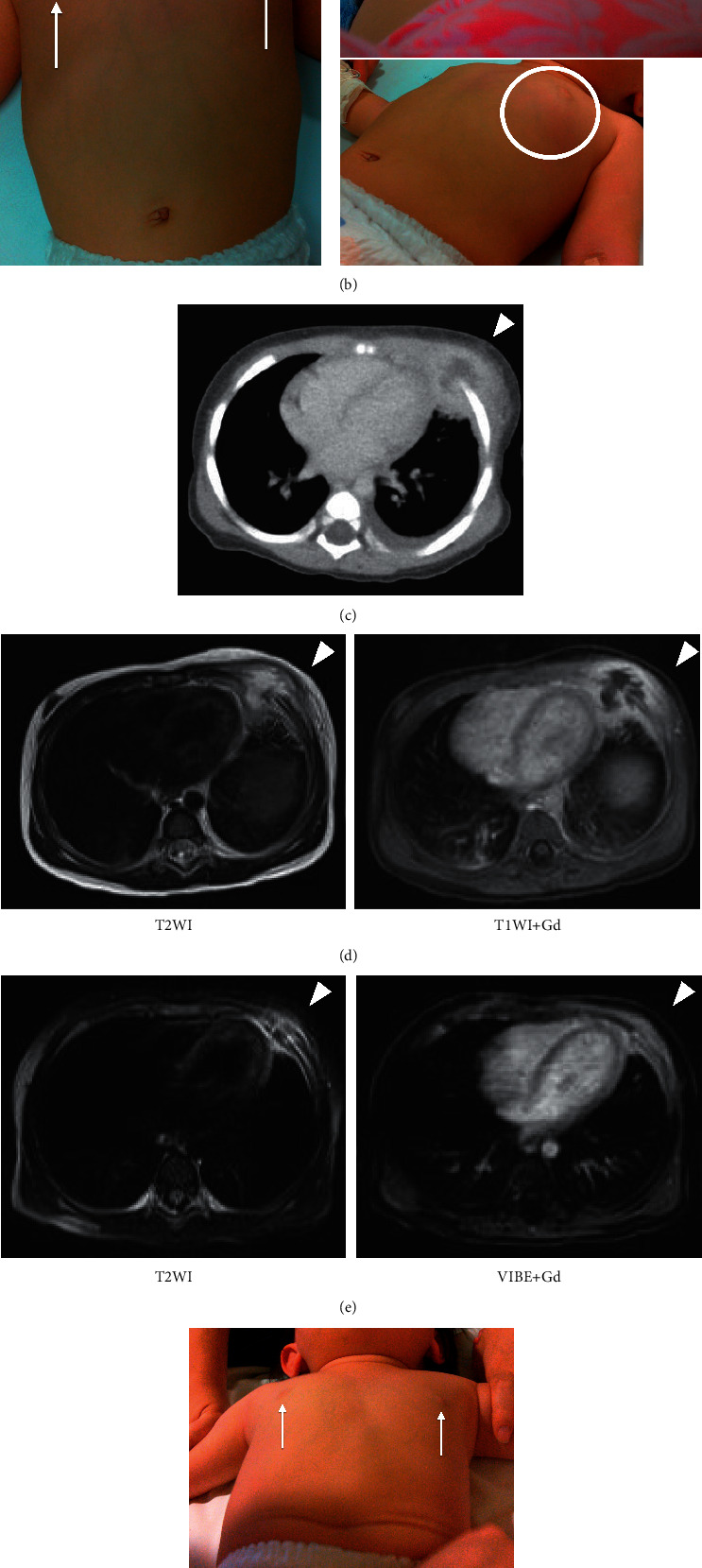
(a) Chest X-ray at admission. The permeability in the left middle and lower lung fields is decreased. The left diaphragm is elevated and the left costophrenic angle is dull. (b) Physical findings of the left anterior chest at the time of diagnosis. The left anterior chest is swollen without redness, and the nipple on the affected side is displaced to the upper left. (c) Initial enhanced chest computed tomography (CT) scan on the second day of admission. A low absorption lesion with a contrast effect is detected around the fifth rib, which suggested a chest wall abscess. Left pleural effusion with low volume is detected. (d) T2-weighted and T1-weighted + gadolinium (Gd) chest magnetic resonance imaging (MRI) on the 3rd day of admission. A high signal on T2-weighted images was observed from the anterior part of the left fifth rib to the transitional part of the costal cartilage. (e) T2-weighted and gadolinium-enhanced chest MRI on the 15th day of admission. The size of the abscess around the left fifth rib is greatly reduced. A contrast-enhanced area can be seen from the anterior part of the left fifth rib to the transitional part of the costal cartilage, resulting in the diagnosis of left fifth rib osteomyelitis. (f) Physical findings of the left anterior chest at the time of after 38 days of treatment. Swelling around the left fifth rib and papillary deviation are improving. (g) T2-weighted and gadolinium-enhanced chest MRI on after 38 days of treatment. The contrast area of the anterior portion of the fifth rib to the costochondral transition of the ribs is also reduced.

A previously healthy one-year-old girl was admitted to our hospital with a fever lasting for three days. She had no medical history, and her vaccination status was satisfactory for her age. There were no apparent epidemics nor any known contact with infected people at her nursery schools. At the time of admission, her body temperature was 39.2°C, heart rate was 169 beats/min, respiratory rate was 28 breaths/min, blood pressure was 102/68 mmHg, and oxygen saturation was 98% in room air. Her chest sounds were clear, and there was pharyngeal redness. A laboratory test showed a white blood cell count of 21,290/*μ*L with 66% neutrophils and a C-reactive protein concentration of 9.9 mg/dL. Urinalysis result showed no urinary tract infection. Chest X-ray on the day of admission showed a cardiothoracic ratio of 55%; the left costophrenic angle was dull, and permeability of the left middle and lower lung fields was decreased (Figure 1(a)). Therefore, she was diagnosed with pneumonia and antibiotic therapy was started with cefotaxime.

On the second day of admission, methicillin-sensitive *Staphylococcus aureus* was detected in blood culture, and the antibiotic was changed to cefazolin (150 mg/kg/day). A physical examination revealed slight swelling on the left anterior chest; her mother had been unaware of the swelling before admission (Figure 1(b)). We suspected that her swollen chest was the cause of the fever, and thus an enhanced chest computed tomography (CT) scan was performed. This scan showed a low absorption lesion centered on the left fifth costal cartilage junction, with adjacent soft tissue swelling and ring enhancement. Additionally, mild left pleural effusion was detected (Figure 1(c)). These findings led to the diagnosis of left anterior chest wall abscess and suspected rib osteomyelitis. On the third day of admission, a contrast-enhanced magnetic resonance imaging (MRI) was performed in order to diagnose rib osteomyelitis. MRI findings showed a 30-mm-large mass in the costochondral transition of the left fifth rib, extending across the ribs to the chest wall and thoracic cavity (Figure 1(d)). In the center of the mass, there was an irregular contrast-enhancing area that extended into the left thoracic cavity and was accompanied by pleural thickening. These results supported the presence of a left anterior chest wall abscess which might have developed from osteomyelitis at the costochondral transition but we were not able to identify osteomyelitis at this time because of the strong signal from the abscess. No methicillin-sensitive *Staphylococcus aureus* was detected in a repeated blood culture after starting cefazolin. On the ninth day of admission, CT scan showed that the abscess had greatly improved. Thus, no surgical treatment was required. On the 15th day of hospitalization, contrast MRI was performed to evaluate for osteomyelitis (Figure 1(e)). The MRI showed that the abscess around the left fifth rib had markedly shrunk, and the swelling in the same area had decreased. At the same time, a contrast area was observed in the anterior part of the left fifth rib to the costochondral transition, leading to the diagnosis of osteomyelitis of the left fifth rib and abscess of the left anterior chest wall. She was treated with cefazolin intravenously for two weeks, and then, this was switched to oral cephalexin (120 mg/kg/day). Subsequently, she was discharged on the 24^th^ day of admission and continued to take cephalexin. Antibiotic therapy was administered for six weeks. Follow-up MRI at 38 days after starting treatment showed that the inflammation around the left fifth rib had improved, and no recurrence was detected (Figures 1(f) and 1(g)).

We evaluated her immune function during her hospitalization which showed no immunological abnormality.

## 3. Discussion

Rib osteomyelitis in children is an extremely rare disease, accounting for approximately 1% of childhood osteomyelitis [1, 2]. While pediatric osteomyelitis in other areas of the body typically occurs during puberty, osteomyelitis of the ribs typically occurs during infancy [2]. Thus, it is difficult for patients to explain their symptoms. In this case, the patient's mother did not notice chest swelling initially, and neither did the attending physicians. The general presentation of rib osteomyelitis has been reported as fever, chest or back pain, and an abscess [1]; however, other articles have reported that such findings are not always presented—instead, patients may display nonspecific clinical features [3, 4]. Because of these nonspecific symptoms, the lack of local findings (such as redness and swelling), and the indolent lesion that often occurs in cases of rib osteomyelitis [3], it is difficult to diagnose this disease early. This may lead to delays in treatment initiation compared with osteomyelitis affecting other parts, such as the long bones [2–4].

Regarding pathogenesis of osteomyelitis, once the bacterial foci are established, phagocytes travel to the site, producing inflammatory exudate [5], forming abscesses, and impacting multiple regions including the bone marrow, cortex, periosteum, and surrounding soft tissues [6]. Our case is also assumed to have developed a chest wall abscess after the onset of osteomyelitis of the ribs because of the complication of bacteremia which established bacterial foci and produced inflammation to spread out to the surrounding tissues, although from the initial MRI image findings, we were not able to diagnose rib osteomyelitis due to the strong signal from the abscess. However, we followed up on the image after the improvement of the abscess; thus, we were able to diagnose the rib osteomyelitis and administer the appropriate term of antibiotics.

In the case of hematogenous osteomyelitis, the most common causative organism is *Staphylococcus aureus*, for which antibiotic therapy alone is sufficient [2]. Although surgical drainage is required when pus revel from the subperiosteal space or metaphysis [5], this procedure is highly invasive for infants. Existing data show no clear evidence of the efficacy of surgical intervention for osteomyelitis [1]. If acute osteomyelitis is diagnosed at a sufficiently early stage, antibiotic treatment alone is sufficient in most cases [1]. A previous case report and literature review found that many cases of rib osteomyelitis required surgical drainage [4] and surgical debridement was required as the optimal treatment for cases of osteomyelitis associated with contiguous infections, or chronic osteomyelitis [4, 5, 7]. Our case was diagnosed early and treatment response was good; thus, surgical drainage was avoided.

This case was typical of rib osteomyelitis in terms of age and site of onset, but the diagnosis was difficult at the time of admission because of the lack of specific symptoms. The clinical symptoms of rib osteomyelitis are less severe than those of typical osteomyelitis in other parts of the body; as such, for early diagnosis in infants, it is important to examine the patient carefully for any subtle abnormalities such as swelling or masses. In our case, it was initially difficult to evaluate the presence of osteomyelitis due to the presence of an abscess, but after the abscess shrank in response to treatment, we were able to diagnose osteomyelitis via MRI. Owing to this early diagnosis, our patient's clinical and imaging symptoms improved with antimicrobial therapy alone, and treatment was completed without the need for surgical intervention.

## Data Availability

The data used to support the findings of this study are included within the article.
